# The Human Microbiomes in Pancreatic Cancer: Towards Evidence-Based Manipulation Strategies?

**DOI:** 10.3390/ijms22189914

**Published:** 2021-09-14

**Authors:** Giovanni Brandi, Silvia Turroni, Florencia McAllister, Giorgio Frega

**Affiliations:** 1 Department of Experimental, Diagnostic and Specialty Medicine, Sant’Orsola-Malpighi Hospital, University of Bologna, 40138 Bologna, Italy; 2 Medical Oncology, IRCCS Azienda Ospedaliero-Universitaria di Bologna, 40138 Bologna, Italy; 3 Unit of Microbiome Science and Biotechnology, Department of Pharmacy and Biotechnology, University of Bologna, 40126 Bologna, Italy; silvia.turroni@unibo.it; 4 Department of Gastrointestinal Medical Oncology, The University of Texas MD Anderson Cancer Center, Houston, TX 77030, USA; FMcAllister@mdanderson.org; 5 Department of Clinical Cancer Prevention, The University of Texas MD Anderson Cancer Center, Houston, TX 77030, USA

**Keywords:** pancreatic cancer, microbiome, immuno-oncology, tumor-targeting bacteria

## Abstract

Recent pieces of evidence have emerged on the relevance of microorganisms in modulating responses to anticancer treatments and reshaping the tumor-immune microenvironment. On the one hand, many studies have addressed the role of the gut microbiota, providing interesting correlative findings with respect to etiopathogenesis and treatment responses. On the other hand, intra-tumoral bacteria are being recognized as intrinsic and essential components of the cancer microenvironment, able to promote a plethora of tumor-related aspects from cancer growth to resistance to chemotherapy. These elements will be probably more and more valuable in the coming years in early diagnosis and risk stratification. Furthermore, microbial-targeted intervention strategies may be used as adjuvants to current therapies to improve therapeutic responses and overall survival. This review focuses on new insights and therapeutic approaches that are dawning against pancreatic cancer: a neoplasm that arises in a central metabolic “hub” interfaced between the gut and the host.

## 1. Introduction

Pancreatic cancer (PC) is a malignancy with a dismal prognosis and a growing incidence rate. Despite enormous efforts to develop new therapeutic strategies, progress remains limited. In parallel, the crucial role of microorganisms in cancer etiopathogenesis and treatment response is surfacing. The low impact of immunotherapeutic strategies on PC [1] did not make this neoplasm an ideal model to evaluate the effect of the intestinal microbiota on treatment outcomes and survival. Despite that, more detailed insights into the role of diet, microbiota, and intra-tumoral bacteria will likely set the stage for the advent of innovative treatment strategies.

In this review, after a summary of the main structural features in health and disease of the most studied human microbiome, i.e., the gut microbiome, we discuss its potential role in influencing PC progression. Then, we comment on recent evidence available on other host-associated microbial communities potentially involved/altered in PC, namely oral and intra-tumoral microbiomes. Based on this, we discuss possible therapeutic strategies using microbiomes/microorganisms as a target/tool to modulate tumor growth and immune response, therefore improving therapeutic responses and prolonging patient survival.

## 2. The Human Gut Microbiome: A Major Homeostasis Player 

The human body is colonized by diverse, abundant communities of microorganisms, collectively referred to as the microbiota [2]. Most of them, mainly bacteria but also fungi and viruses, reside in the gut and are known to deeply influence our physiology, from vitamin synthesis and extraction of energy from indigestible carbohydrates, to modulation of the immune, endocrine and nervous systems [3,4,5,6]. This is made possible by the wide and diverse range of bioactive small molecules produced or contributed by gut microbes, e.g., short-chain fatty acids (SCFAs) as byproducts of fiber fermentation, which can enter the bloodstream and influence extra-intestinal organs [7]. It should also be remembered that the gut microbiota has the potential to metabolize xenobiotics, converting them into potentially active, inactive or even toxic metabolites [8], which supports its influential role on pharmacokinetics and pharmacodynamics, hence the efficacy and toxicity of therapies, as already demonstrated in different contexts [9,10].

Gut microbiota profiles are highly individualized and dynamic, being continuously shaped by internal and external exposures. Population-based metagenomics studies are consistent in showing that environment dominates over host genetics in microbiota modeling, with the term “environment” being understood as a combination of diet and lifestyle factors, including socio-economic and cultural aspects, physical activity, household sharing, environmental exposure, health care, etc. [11,12,13]. Among these, diet is definitely recognized as a pivotal determinant of gut microbiota structure and function, able to sustain homeostasis but also to contribute to disease susceptibility, by interfering in the microbiota-host signaling [14].

Unbalanced, i.e., dysbiotic profiles of the microbiota have been found to date in a multitude of disorders, ranging from gastrointestinal, metabolic, autoimmune, hepatic, respiratory, cardiovascular to neurological, psychiatric and even oncological ones, and are supposed to variously contribute to their onset and progression [2,15,16,17,18]. According to the “common ground hypothesis”, it is likely that endogenous and/or environmental factors trigger an increase in intestinal permeability (i.e., leaky gut) or mucosal inflammation, directly or through selective pressures on the gut microbiota. This process would favor the expansion of opportunistic microbes and their transition to pathobionts, with downstream induction of pathogenic morphological and functional changes [15]. Such disease-promoting microbiota layouts are generally featured by reduced diversity, loss of beneficial microbes (mainly SCFA producers) and/or enrichment of strict or opportunistic pathogens [19]. Recent studies also stress the relevance of reconstructing personal dysbiotic trajectories over time to discriminate between different types of dysbiosis (e.g., “locative” vs. “volatile”), to be treated differentially in evidence-based personalized intervention strategies [20,21]. 

## 3. The Role of the Gut Microbiome in Pancreatic Cancer

Even though cancer is perceived as a genetic disease, 13% of human malignancies, and probably a higher percentage in other animal species, are more or less strictly dependent on microorganisms in their etiopathogenesis [22,23]. 

Among malignancies, PC is one of the most complex and peculiar. Years of research on this neoplasm have led to minimal improvements in patient treatment and survival. This has resulted in a progressive increase in the impact of this tumor on the worldwide cancer-specific mortality, accompanied by an increase in its incidence. It is actually expected that by 2030 PC will be the second leading cause of cancer-related deaths [24].

From a histological point of view, it consists of ductal epithelial cells, acinar exocrine cells, endocrine cells confined in the islets of Langerhans, vascular/stromal cells and immune cells. Unlike other areas of the gastrointestinal tract, a proper pancreatic stem cell compartment has never been identified. However, the cells that constitute these acinar glands, even if terminally differentiated, are able to regenerate in the case of chronic injury leading to gland renewal, and could also face a metaplastic process called acinar-to-ductal trans-differentiation (ADTD) that accounts for a minority of ductal metaplasia with typical mucinous features [25,26]. This phenomenon has been described in vitro [27] and demonstrated in vivo by lineage tracing using the Cre-loxP-based system [25]. At least three precancerous pancreatic lesions have been identified to date: pancreatic intraepithelial neoplasia (PanIN), intraductal pancreatic mucinous neoplasm (IPMN) and mucinous cystic neoplasm (MCN) [28]. Pancreatic intraepithelial neoplasia (PanIN) is recognized as the most common cancer precursor pathway and its pathological evidence is significantly higher in the pancreas of patients with pancreatic ductal adenocarcinoma (PDAC) than those with benign conditions [29].

Studies have recently uncovered the key role of microbes in pancreatic carcinogenesis as well as their influence in modulating the activity of chemotherapies and immunotherapies used for numerous malignancies [30,31,32]. Despite the evidence of an immune-dependent tumor-promoting effect, conversely from colon cancer, a clear correlation between the microbiota and the development of specific preneoplastic pancreatic lesions has not been described.

**Figure 1:**
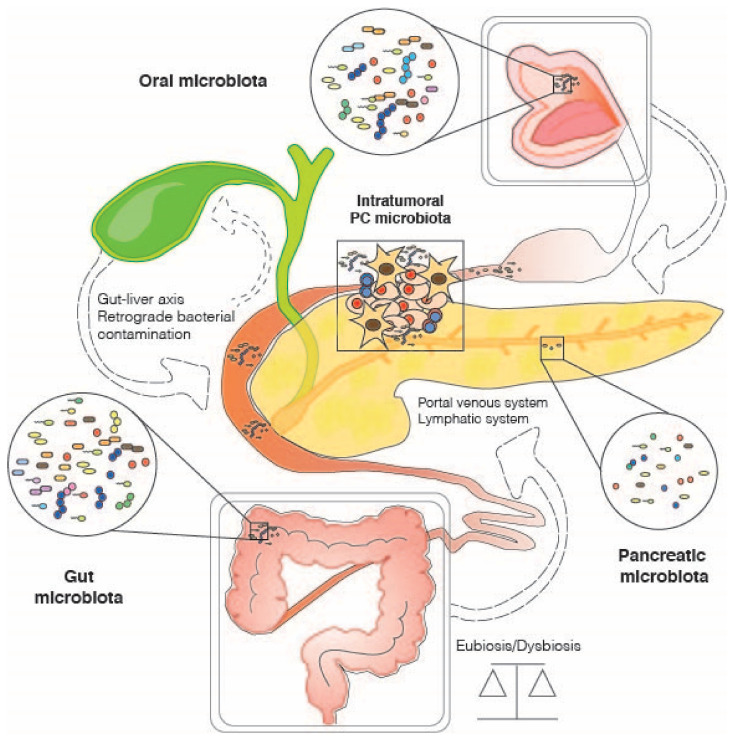
Potential bacterial interchange routes between gut, oral and intra-tumoral PC-associated microbiota.

With specific regard to PC, the “microbiota-cancer axis” probably relies, to varying degrees, on several other microbiota-modulated spindles, such as the microbiota-immune-inflammatory axis [31], the microbiota-brain axis [33,34] and the microbiota-liver axis [35,36]. The latter could be differently dysregulated in subsequent steps of pancreatic carcinogenesis (Figure 1). In addition, multiple environmental stressors, already known for their involvement in increasing the risk of PDAC, such as impaired nutrition, smoking, alcohol abuse, obesity and insulin resistance, can also exert their actions through dysbiosis of the intestinal microbial communities, with ultimately severe repercussions on whole-body health [37,38,39]. Some researchers are elegantly shedding light on the potential microbiota-mediated mechanisms of action employed by these stressors [40,41,42]. For example, smoking is assumed to have a direct or indirect impact on the microbiome through immunosuppression and biofilm formation, potentially favoring harmful pro-inflammatory taxa [30]. Among the major microbial components involved, gut-derived microbe-associated molecular patterns (MAMPs), such as lipopolysaccharide (LPS) and lipoteichoic acid (surface components of Gram-negative and Gram-positive bacteria, respectively), must certainly be mentioned [43,44]. They can in fact trigger inflammatory responses through interaction with Toll-like receptors, whose expression has been found to be increased in the PDAC microenvironment [45,46]. Furthermore, deoxycholic acid (DCA), a secondary bile acid resulting from the metabolic conversion of cholic acid by intestinal bacteria, has been shown to promote carcinogenesis by accelerating senescence-associated secretory phenotypes (with consequent immunosuppression) and by inducing DNA damage and genomic instability [47,48]. Not least, DCA can activate the epidermal growth factor receptor (EGFR) and promote the release of its ligand, amphiregulin, which has been shown to participate in DCA-induced EGFR and STAT3 signaling, and PDAC tumorigenicity [49].

It is, therefore, not surprising that the PC-associated gut microbiota is typically enriched in pro-inflammatory genera, especially Gram-negative bacteria belonging to the phylum Proteobacteria, including *Escherichia*, *Erwinia*, *Proteus* and *Klebsiella*. Such an increase has been reported, for example, in fecal specimens of PDAC patients compared to the normal population in the U.S. [31], as well as in a Chinese cohort of PC patients (compared to matched healthy subjects), together with the reduction in beneficial microbes and butyrate producers, such as bifidobacteria, *Coprococcus* and *Anaerostipes* [50]. In addition, these reports have shown increased proportions of Verrucomicrobia members (well-known mucus degraders) and *Veillonella* (a lactate user) in cancer patients [31,37]. More recently, in an Israeli cohort, the authors confirmed most of these observations, finding that the PC-associated gut microbiome was enriched in *Veillonellaceae*, as well as in *Akkermansia* and *Odoribacter*, while depleted in beneficial SCFA-producing families, i.e., *Lachnospiraceae* and *Ruminococcaceae* [51]. As previously discussed [19], the reduction in the latter may represent a non-specific, shared response to diseases, probably related to the occurrence of increased oxidative stress. On the other hand, the enrichment of Verrucomicrobia and *Veillonellaceae* could be a distinctive signature, robust to geography. As for the former, it is worth mentioning that *Akkermansia* (the main genus of Verrucomicrobia) is widely recognized as beneficial in the context of metabolic disorders [52] but has also been shown to exacerbate the symptoms of multiple sclerosis, possibly through induction of pro-inflammatory T lymphocyte responses and impairment of barrier function [53]. Although we are aware that these are completely different disorders, we are trying to speculate that its overrepresentation could be related to an increased translocation and inflammatory tone, even in PC. 

As anticipated above, this PC-associated gut microbiome may foster malignant progression via several mechanisms, including the induction of innate and adaptive immunosuppression. Pushalkar et al. [31] in fact found that germ-free mice were protected against PDAC progression, while fecal microbiota transplantation (FMT) from PDAC-bearing mice not only reversed this protection but accelerated tumorigenesis, preventing Th1 differentiation of CD4+ T cells and CD8+ T cell activation. However, it remains extremely hard to define a direct causal relationship between PC-enriched microbial taxa and cancer development/progression. In other words, it is challenging to establish whether some risk factors (in concert with individual genetic predisposition) can actually induce a distinctive dysbiosis and, consequently, foster the PC development or, conversely, they all act at the same time, without a temporal and pathogenic sequential relationship between them. 

## 4. Other Host-Associated Microbiomes and Pancreatic Cancer: Oral and Intra-Tumoral Microbial Communities

### 4.1. Oral Microbiome

Not only does the gut microbiota appear to be related to the occurrence of PC but also some oral bacteria (e.g., *Fusobacterium*, *Porphyromonas gingivalis*, *Neisseria elongata* and *Streptococcus mitis*) have been shown to confer augmented susceptibility to this neoplasm [44]. Recent work has even reported the presence of typically oral bacteria (e.g., *Granulicatella adiacens* and the likely “oncobacterium”, *Fusobacterium nucleatum*) in the cyst fluid from intraductal papillary mucinous neoplasm (IPMN), possibly as a consequence of inflammation and bacterial translocation and/or in some way related to the functional similarities between pancreas and salivary glands [54]. According to the same authors, high-grade cystic lesions and full malignant lesions show higher bacterial quantity (i.e., 16S rDNA copies) than low-grade IPMN. An analogous result was obtained by comparing the bacterial DNA amount in malignant lesions with that in normal pancreatic tissue [31].

Oral dysbiosis in PC constitutes a paradigmatic example. Several studies have described the impact of personal behaviors, such as smoking or poor dental hygiene, in inducing alterations of the oral microbic communities. Others have reported a correlative relationship between dysbiotic events and the risk of cancer development and mortality [55]. In particular, *P. gingivalis*, a Gram-negative anaerobic pathogen, has been linked to a high risk of developing PC. A high abundance of serum antibodies against this bacterium doubles the odds ratio for PC occurrence [56]. At the molecular level, *P. gingivalis* secretes the enzyme peptidyl-arginine deiminase, capable of degrading arginine, which might lead to *p53* and *K-ras* mutations, associated with poor prognosis of PC patients [57]. Smoking habits appear to foster *P. gingivalis* infectivity and suppress the host’s systemic IgG response against this species [58,59]. Untangling and defining the relative impact of microbiota-dependent carcinogenesis is challenging. More directly, it is smoking habits that affect both cancer risk and oral dysbiosis or, conversely, a dominant role of smoking-related carcinogenesis is to be referred to this microorganism itself. Eventually, cigarette smoke could also act along opposite trajectories, on the one hand increasing the risk due to its chemical carcinogens and on the other hand lowering it, by impairing the humoral response against *P. gingivalis*.

### 4.2. Intratumor Microbiome

With specific regard to the structure of the intra-tumoral microbiota, the phylum Proteobacteria was found to prevail over the others in most cases of low-grade lesions (with particularly, overrepresentation of *Enterobacteriaceae* members, such as *Escherichia*/*Shigella* and *Klebsiella*, as well as *Pasteurellaceae* and *Methylobacteriaceae*), while the cancer-related microbiota appears to be more diversified with a dominance of either Proteobacteria or Firmicutes [54]. According to recent estimates, up to 25% of the pancreatic tumor microbiota could directly originate from gut microbial communities through translocation but, in any case, be shaped by these through indirect mechanisms, such as modulation of immune function [60]. As for the potential bias represented by previous endoscopic procedures, only a few articles have taken it into consideration. Among these, Gaiser et al. reported the contribution of these procedures in increasing the bacterial load within the lesion, while antibiotic administration and proton-pump inhibitor usage do not seem to exert a relevant impact on the latter [54]. Recently, the mycobiota (i.e., the set of fungal communities) has also been implicated in tumorigenesis. According to Aykut et al. [61], fungi can migrate from the gut lumen to the pancreas, where they promote PDAC by triggering the complement cascade through mannose-binding lectin activation. Among these, a leading role has been attributed to *Malassezia*, specifically *Malassezia globosa*, which was found to be particularly abundant in human PDAC and capable of accelerating oncogenesis in mouse models based on the potent anti-tumoral activity of anti-fungals.

Although the advent of immunotherapy has completely overhauled the way cancer is treated, the results from single agents or combined immunotherapeutics are daunting in PC [62]. Unfortunately, the objective response rate is also discouraging compared to other cancers, besides an extreme paucity of long-term responders. A plausible explanation for the limited efficacy can arise from the abundant fibrotic microenvironment. The latter can exert a passive role by limiting the drug delivery within the tumor and an active role by releasing specific chemokines (i.e., CXCL12) [63]. Another feature of PDAC is the immunosuppressive microenvironment. According to a recent study, it seems plausible to modulate the intra-tumoral microbial composition and, as a direct consequence, the tumoral immune microenvironment by modifying the gut microbiota by FMT [60]. Moreover, the same authors reported that higher intra-tumoral α-diversity (i.e., intra-sample diversity, generally referring to the richness and/or evenness of a given microbial ecosystem) correlated with long-term survival in two retrospective patient cohorts, and identified peculiar favorable intra-tumoral microbial signatures, including *Saccharopolyspora*, *Pseudoxanthomonas*, *Streptomyces* and *Bacillus clausii* [60]. These taxa may contribute to the antitumor immune response by favoring the recruitment and activation of CD8+ T cells, with IFN-gamma overproduction, ultimately affecting the natural history and survival of PDAC. Among intra-tumoral microbes, Gammaproteobacteria members were found to mediate the enzymatic degradation of gemcitabine, currently the cardinal chemotherapeutic agent in PDAC treatment, into an inactive metabolite (2′,2′-difluorodeoxyuridine) [30]. The administration of antibiotics appears to be able to increase the efficacy of gemcitabine in terms of tumor response, when Gammaproteobacteria-colonized colon tumors were subcutaneously grown in mice [30]. Recent clinical studies that have looked at the effect of antibiotics in PDAC have demonstrated a beneficial effect, specifically on patients that have received gemcitabine-based therapies [64,65].

Please see Table 1 for a summary of available studies on host-associated microbiomes (gut, oral and intra-tumoral) and PC, in both animal models and humans.

## 5. Potential Therapeutic Strategies: Diet, Probiotics, Fecal Microbiota Transplantation, and Tumor-Targeting Bacteria 

**Figure 2:**
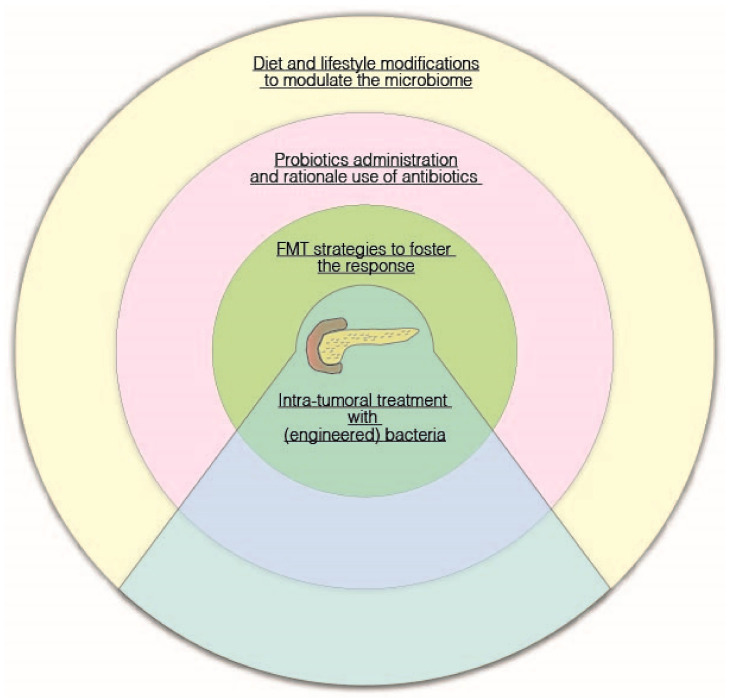
A figurative “dartboard” on potential microbiota-related therapeutic strategies in pancreatic cancer.

As discussed above, available evidence from animal models and human studies suggests that host-associated microbiomes/microorganisms might be useful as a predictor of patient outcomes, and therefore their modulation could represent a promising adjunct to current therapies, to change the tumor microenvironment and sensitize tumors to therapeutics (Figure 2).

In recent years, several clinical trials have been specifically designed to investigate the therapeutic potential of the gut microbiome manipulation directly in cancer patients, through nutritional intervention, administration of probiotics or FMT [68]. Below, we discuss the clinical trials registered over the past six years, aimed at evaluating the impact of microbiome modulation tools in PC patients (see also Table 2).

For several obvious reasons, such as safety profile, cost and accessibility, the diet represents a simple approach to assess the implications of microbiome manipulation and downstream immune responses. With specific regard to PC, for example, one clinical trial is currently recruiting patients with the aim of evaluating the effect of ketogenic diet in metastatic PC during chemotherapy (NCT04631445), while another plans to examine the efficacy of a 12-week multidisciplinary rehabilitation program, consisting of dietary counseling and group education sessions, for survivors of PC, as well as of other cancers of the upper gastrointestinal tract, namely cancers of the esophagus, stomach and liver (NCT03958019). However, to the best of the authors’ knowledge, neither of these, nor of the older ones, included assessment of the gut microbiota profile by any technique.

On the other hand, the administration of probiotics is a more feasible approach in clinical practice, due to the sometimes-modest effects of nutritional interventions and the difficult of enforcing and monitoring patient compliance. However, as far as we know, no clinical trials with probiotics in PC have been conducted. Without prejudice to the validity of traditional probiotics, although not to be considered absolute as recently discussed [38,69], it should be emphasized that the accumulating knowledge of the human microbiome, accelerated by massive sequencing, is dramatically expanding the range of microorganisms with potential health benefits in the context of specific diseases. Such microorganisms, referred to as next-generation probiotics or live biotherapeutics [70], include, for example, SCFA producers (e.g., *Faecalibacterium prausnitzii*, proposed for the treatment of inflammatory bowel disease and other inflammation-based disorders) [71], *Akkermansia* (for obesity and related complications) [52], and *Bacteroides* species, such as *Bacteroides xylanisolvens* and *Bacteroides ovatus*, both associated with increased levels of Thomsen-Friedenreich antigen-specific antibodies and potentially with improved cancer immunosurveillance [72,73]. It has also been thought to engineer GRAS (generally recognized as safe) organisms or commensals as a delivery vehicle for bioactive molecules or to express certain functionality, as is the case with *Lactococcus lactis* and elafin [74], trefoil factor 1 [75] and IL-10 [76], *B. ovatus* and IL-2 [77] or TGF-beta 1 [78], and *Escherichia coli* Nissle 1917, which has been modified to bind to the surface of cancerous cells and secrete myrosinase, an enzyme capable of converting glucosinolates to isothiocyanates, such as sulforaphane, a molecule with known antitumor activities [79]. As for PC, based on gut microbiome evidence, it is reasonable to speculate that SCFA producers might also be useful in this context, mainly because of their role in strengthening intestinal barrier integrity, therefore preventing translocation of microbial components and microbes, and their antimicrobial activity, including the control of fungal growth [80,81]. On the other hand, the data on the tumor microbiome suggest the possibility of using *B. clausii*, a probiotic currently available in the marketplace, as well as the other microorganisms identified as potential signatures of long-term survivorship, although their direct functional contribution to cancer progression is not yet defined [60,82]. Keeping in mind the relevance of immune activation in the tumor milieu, microbial engineering strategies aimed at the on-site recruitment of CD8+ T cells through the production of IFN-gamma or interfering with the lectin pathway of the complement cascade, should be investigated as well. Although bacteriotherapy for PC remains largely unexplored, synthetic biology holds great promise for specifically targeting tumors, actively penetrating tissues and controllably inducing cytotoxicity [83]. Not least, to enhance therapeutic efficacy, antibiotic treatments that decrease the number of Gammaproteobacteria capable of inactivating gemcitabine or the use of inhibitors specific for the microbial enzyme involved in this inactivation may also deserve consideration.

FMT is certainly the most direct means of manipulating the intestinal microbiota and represents an immense therapeutic opportunity for patients with PC, where there are still few viable options. FMT preparations can be administered to patients orally, in the form of freeze-dried or frozen pills, or by colon or gastroscopy. Data are being accumulated in relation to several cancers while only one study has just been registered (and is not yet recruiting), with the primary objective of assessing the safety, tolerability and feasibility of FMT in resectable PDAC patients (NCT04975217). Patients will undergo FMT during colonoscopy and receive FMT capsules via *os* once a week for 4 weeks, then undergo surgery to remove the tumor. They will be followed up to 6 months after surgery to determine immunological/molecular changes, and to assess changes in the gut, oral and intra-tumoral microbiome. As discussed above for preclinical data [60], FMT is expected to alter human microbiomes, including the intra-tumoral one, activating the immune system and inducing antitumor responses, with microbiome-dependent CD8+ T cell activation and decreased tumor infiltration by Tregs.

## 6. Conclusions

Although being aware of the urgent need to conduct further studies to disentangle the contribution of the human microbiomes to PC and validate their potential for early diagnosis and risk stratification, we believe that their manipulation represents an attractive and promising way to modulate tumor immunosuppression and growth, to ultimately improve therapy responses and prolong survival. Given the anatomical position and physiological function of the pancreas, it is easy to speculate on the potential pivotal role of nutrition and the gut microbiota in the neoplastic lesions originating in this organ. Diet modulation, microbiota reshaping, alongside with intra-tumoral bacteria-mediated innovative therapies could probably constitute a novel attractive strategy of treatment for PC patients. 

## Figures and Tables

**Table 1:** List of relevant studies on alterations in host-associated microbiomes and pancreatic cancer. For each study, the design, the method of microbiota analysis, the main results and the reference are reported. The following microbiomes were considered: gut, oral and intra-tumoral.

Ref.	Study Design (Subjects and Samples)	Methods of Microbiota Analysis	Main Results
**Gut Microbiome**			
Ren et al., 2017 [50]	Prospective cohort study on stool samples from: -85 PC patients -57 matched healthy controls	16S rRNA gene sequencing (Illumina MiSeq platform)	PC patients showed reduced gut microbiota diversity, increased proportions of some pathogens and LPS-producing bacteria (*Veillonella*, *Klebsiella*, *Selenomonas*, *Prevotella*, *Hallella* and *Enterobacter*), and reduced amounts of beneficial taxa, such as bifidobacteria and butyrate producers (*Coprococcus*, *Flavonifractor*, *Anaerostipes* and *Clostridium* cluster IV members). The microbial community in obstruction cases was distinct from the unobstructed cases. Through inferred metagenomics, the authors found enrichment in leucine and LPS biosynthesis in PC. Based on 40 genera associated with PC using LDA selection, a high classification power with AUC of 0.842 was achieved.
Pushalkar et al., 2018 [31]	Prospective cohort study on: -PDAC patients (33 rectal swabs and 12 tissue samples) -Matched healthy volunteers (31 rectal swabs and 5 tissue samples) -Murine models (WT, KC and KPC mice)	16S rRNA gene sequencing (Illumina MiSeq platform) FISH qPCR for total bacterial DNA in pancreatic tissues and feces	The cancerous pancreas showed a more abundant microbiome than normal tissue in both mice and humans, with distinct bacterial signatures from the gut. The relative abundance of Proteobacteria, Actinobacteria, Fusobacteria and Verrucomicrobia was greater in the gut of PDAC patients than in healthy controls. Proteobacteria members were also enriched in the intratumor microbiome and associated with advanced disease. Germ-free mice were protected against PDAC, while FMT from PDAC-bearing mice reversed that protection. Microbiota ablation by antibiotics was associated with immunogenic reprogramming of the tumor microenvironment, reduction in myeloid-derived suppressor cells, increase in M1 macrophage differentiation, Th1 differentiation of CD4+ T cells and CD8+ T cell activation.
Half et al., 2019 [51]	Cohort study on stool samples of: -30 patients with PDAC -6 patients with precancerous lesions -16 patients with non-alcoholic fatty liver disease -13 healthy subjects	16S rRNA gene sequencing (Illumina MiSeq platform)	PC patients showed reduced proportions of health-associated bacterial families (*Clostridiaceae*, *Lachnospiraceae* and *Ruminococcaceae*), and increased amounts of *Veillonellaceae*, *Akkermansia* and *Odoribacter*. These microbial signatures were distinct from those of common PC comorbidities, namely bile duct obstruction and liver damage. Based on the discriminating features between PC patients and healthy subjects, a high classification power with AUC of 82.5% was achieved. However, only a few microbial alterations were present in patients with precancerous pancreatic lesions, limiting the potential for early diagnosis.
**Oral Microbiome**			
Farrell et al., 2012 [66]	PRoBE study with microbial profiling of saliva of: -10 resectable PC patients -10 matched healthy controls Validation of bacterial candidates in an independent cohort of 28 resectable PC, 28 matched healthy control and 27 chronic pancreatitis samples	16S rRNA-based oligonucleotide microarray, the Human Oral Microbe Identification Microarray (HOMIM) qPCR	PC patients showed reduced levels of *Neisseria elongata* and *Streptococcus mitis*, and increased levels of *Granulicatella adiacens*. *N. elongata* and *S. mitis* were validated using independent samples. Based on the combination of these two bacterial candidates, a high classification power with AUC of 0.9 was achieved.
Michaud et al., 2013 [56]	Prospective cohort study evaluating antibodies to oral bacteria in pre-diagnosis blood samples from: -405 PC cases -416 matched controls	Immunoblot array using a pre-selected panel of whole-cell formalin fixed bacterial antigens	High levels of antibodies against *Porphyromonas gingivalis* were associated with a two-fold higher risk of PC. High levels of antibodies against commensal bacteria might be associated with reduced risk of PC.
Wei et al., 2020 [67]	Prospective cohort study on saliva of: -41 PDAC patients -69 healthy individuals	16S rRNA gene sequencing (Illumina NovaSeq platform)	*Streptococcus* and *Leptotrichia* were associated with a higher risk of PDAC, while *Veillonella* and *Neisseria* with a lower risk. *Porphyromonas*, *Fusobacterium* and *Alloprevotella* were enriched in patients with bloating, *Prevotella* in those reporting jaundice, and *Veillonella* in those reporting dark brown urine. *Neisseria* and *Campylobacter* were depleted in patients with diarrhea, and *Alloprevotella* in those reporting vomiting.
**Intratumor Microbiome**			
Geller et al., 2017 [30]	Cohort study on: -113 human PDACs -20 samples from normal human pancreas (from organ donors)	qPCR rRNA FISH Immunohistochemistry using an anti-LPS antibody 16S rRNA gene sequencing (on 65 PDACs, Illumina MiSeq platform)	76% of PDACs were positive for bacteria, mainly Gammaproteobacteria (*Enterobacteriaceae* and *Pseudomonadaceae* families). More bacteria were detected in patients undergoing pancreatic duct instrumentation, suggesting possible retrograde bacterial migration from the duodenum to the pancreas. Bacteria cultured from fresh human PDAC tumors made human colon carcinoma cell lines completely resistant to gemcitabine, suggesting that intratumor bacteria might contribute to the drug resistance of these tumors.
Riquelme et al., 2019 [60]	Cohort study on surgical resected PDACs from: -36 long-term survivors -32 short-term survivors (+9 frozen PDACs) Murine models	16S rRNA gene sequencing (Illumina MiSeq platform) rRNA FISH Immunohistochemistry using an anti-LPS antibody PCR with species-specific primers for *Saccharopolyspora rectivirgula*	Long-term survivors showed higher alpha diversity of the intra-tumoral microbiome and enrichment in *Pseudoxanthomonas*/*Streptomyces*/*Saccharopolyspora*/*Bacillus clausii* (in both discovery and validation cohorts). Based on the combination of these 4 taxa, a high classification power with AUC > 97% was achieved. Through inferred metagenomics, the authors found that long-term survivors were enriched in pathways related to the metabolism of amino acids, xenobiotics, lipids, terpenoids and polyketides, besides other cellular functions. Human-into-mice FMT experiments showed that the gut microbiome can modulate the intra-tumoral microbiome, partly by direct translocation, partly by altering the microbial landscape.

AUC, area under the curve; FISH, fluorescence in situ hybridization; FMT, fecal microbiota transplantation; KC, mice expressing mutant intra-pancreatic *K-ras*; KPC, mice expressing mutant intra-pancreatic *K-ras* and *p53*; LDA, linear discriminant analysis; LPS, lipopolysaccharide; PC, pancreatic cancer; PDAC, pancreatic ductal adenocarcinoma; PRoBE, prospective specimen collection before outcome ascertainment and retrospective blinded evaluation; qPCR, quantitative polymerase chain reaction; WT, wild-type mice.

**Table 2:** Clinical trials registered on ClinicalTrials.gov (as accessed on 7 September 2021) concerning diet and fecal microbiota transplantation (FMT) as adjuvant therapy in pancreatic cancer patients. Search terms included “pancreatic cancer”, in combination with “diet” or “FMT”. Only the trials started no earlier than 2015 were considered. No results were returned when searching for probiotics.

	Title	Status	Results	Condition	Intervention	Location	URL
**Diet**	Diet and Exercise After Pancreatic Cancer (PACE)	Recruiting	No results available	PC	Diet counseling delivered using visual communication	U.S.	https://clinicaltrials.gov/ct2/show/NCT03187028
	Study Evaluating the Ketogenic Diet in Patients with Metastatic Pancreatic Cancer	Recruiting	No results available	Metastatic PDAC	Ketogenic diet	U.S.	https://clinicaltrials.gov/ct2/show/NCT04631445
	Rehabilitation Strategies Following Esophagogastric and Hepatopancreaticobiliary Cancer (RESTORE II)	Not yet recruiting	No results available	PC, esophageal cancer, gastric cancer, liver cancer	RESTORE II program	Ireland	https://ClinicalTrials.gov/show/NCT03958019
	Nutrition Support to Improve Outcomes in Patients with Unresectable Pancreatic Cancer	Active, not recruiting	No results available	PC	Diet with and without nutrition supplementation (Nutrawell Powder or OmegaRich fish oil supplement)	U.S.	https://clinicaltrials.gov/ct2/show/NCT02681601
	Gemcitabine Hydrochloride, Paclitaxel Albumin-Stabilized Nanoparticle Formulation, Metformin Hydrochloride, and a Standardized Dietary Supplement in Treating Patients with Pancreatic Cancer That Cannot be Removed by Surgery	Active, not recruiting	No results available	PC	Dietary supplements (curcumin, vitamin D, vitamin K2, vitamin K1, B6, high-selenium broccoli sprouts, epigallocatechin gallate, L-carnitine, garlic extract, genistein, zinc amino chelate, mixed tocopherols, ascorbic acid, D-limonene)	U.S.	https://clinicaltrials.gov/ct2/show/NCT02336087
	Prevention of Chyle-leak After Major Pancreatic Surgery	Recruiting	No results available	PC with lymph leakage, chyle into mesentery (extravasation)	No-fat diet including medium-chain fatty acids as oil and protein supplements	Finland	https://clinicaltrials.gov/ct2/show/NCT03167814
	Exercise and Nutrition to Improve Pancreatic Outcomes	Recruiting	No results available	PC	Nutritional counseling	U.S.	https://clinicaltrials.gov/ct2/show/NCT03256201
	Early Oral Versus Enteral Nutrition After Pancreatoduodenectomy	Unknown	No results available	PC, duodenal cancer, cholangiocarcinoma, chronic pancreatitis	Oral vs. enteral nutrition	Poland	https://clinicaltrials.gov/ct2/show/NCT01642875
	Ascorbic Acid and Combination Chemotherapy in Treating Patients with Locally Advanced or Recurrent Pancreatic Cancer That Cannot Be Removed by Surgery	Completed	Has results	PC, PDAC	Ascorbic acid as dietary supplement	U.S.	https://clinicaltrials.gov/ct2/show/NCT02896907
	Improving Outcomes in Cancer Patients with a Nutritional and Physical Conditioning Prehabilitation Program	Recruiting	No results available	PC, liver cancer, bile duct cancer, hepatobiliary cancer	A high-protein diet, possibly with a whey-protein supplement	Canada	https://clinicaltrials.gov/ct2/show/NCT03475966
	Enteral Nutrition After Pancreaticoduodenectomy	Completed	No results available	PC, duodenal cancer, ampulla of Vater cancer, cholangiocarcinoma	Enteral diet administered through a nasojejunal tube vs. oral intake	China	https://clinicaltrials.gov/ct2/show/NCT03150615
	High Dose Ascorbic Acid (AA) + Nanoparticle Paclitaxel Protein Bound + Cisplatin + Gemcitabine (AA NABPLAGEM) in Patients Who Have Metastatic Pancreatic Cancer	Withdrawn (regulatory review needed)	No results available	Metastatic PC	Ascorbic acid as dietary supplement	U.S.	https://clinicaltrials.gov/ct2/show/NCT03797443
	Enhancing Fitness Before Pancreatic Surgery (MedEx)	Completed	No results available	PC, chronic pancreatitis	Nutritional supplementation (based on components of the Mediterranean diet)	United Kingdom	https://clinicaltrials.gov/ct2/show/NCT02940067
	Whipple Protein Study (WPS)	Not yet recruiting	No results available	PC, malnutrition	High-protein nutritional supplementation	U.S.	https://clinicaltrials.gov/ct2/show/NCT04306874
	A Study to See Whether a Nutritional Supplement is Beneficial for Patients with Pancreatic Cancer	Withdrawn (change in concept)	No results available	PC	Nutritional supplement	Canada	https://clinicaltrials.gov/ct2/show/NCT02745197
	A Study of the Efficacy of ONS to Reduce Postoperative Complications Associated with Pancreatic Surgery (INSPIRE)	Terminated (Closed due to low enrollment)	No results available	PC, chronic pancreatitis	Dietary counseling with and without oral nutritional supplementation (Ensure Surgical)	U.S.	https://clinicaltrials.gov/ct2/show/NCT03244683
	Zinc Supplements in Lowering Cadmium Levels in Smokers	Completed	No results available	PC, bladder, cervical, esophageal, gastric, head and neck, kidney, liver, lung cancer, leukemia	Dietary supplement (zinc oxide)	U.S.	https://clinicaltrials.gov/ct2/show/NCT00376987
	Effect of Preoperative Immunonutrition in Upper Digestive Tract	Recruiting	No results available	PC, gastric, esophageal cancer	Immunomodulatory oral nutritional supplement (enriched in arginine, nucleotides, omega-3 fatty acids, olive oil polyphenols, L-carnitine, and antioxidants)	Spain	https://clinicaltrials.gov/ct2/show/NCT04027088
	Resistance Training Intervention to Improve Physical Function in Patients with Pancreatic Cancer Receiving Combination Chemotherapy or Have Undergone Surgery, PancStrength Study	Recruiting	No results available	PC, PDAC	Individualized recommendations for daily protein intake and information about healthy protein supplementation during chemotherapy	U.S.	https://clinicaltrials.gov/ct2/show/NCT04837118
	Gemcitabine and Capecitabine with or Without T-ChOS as Adjuvant Therapy for Patients with Resected Pancreatic Cancer (CHIPAC)	Terminated (Poor accrual and change of SOC)	No results available	PC	T-ChOS as dietary supplement (a blend of chit oligosaccharides from shellfish-derived chitin)	Denmark	https://clinicaltrials.gov/ct2/show/NCT02767752
	Effects of Prehabilitation and Early Mobilization for Patients Undergoing Pancreas Surgery (PreMob)	Completed	No results available	PC	Dietary advice	Sweden	https://clinicaltrials.gov/ct2/show/NCT03466593
	Preoperative Prehabilitation for Sarcopenic Patients Prior to Pancreatic Surgery for Cancer (PSOAS)	Not yet recruiting	No results available	PC	Dietary supplement (Oral Impact^®^)	France	https://clinicaltrials.gov/ct2/show/NCT04469504
	Cost Effectiveness of an Intervention in Hospitalized Patients with Disease-related Malnutrition	Recruiting	No results available	PC, acute pancreatitis, IBD, esophagus, gastric, colorectal cancer	Dietary advice, oral nutritional supplementation vs. no explicit intervention	Spain	https://clinicaltrials.gov/ct2/show/NCT04188990
	The Pancreatic and Periampullary Resection Arginine Immunomodulation (PRIMe) Trial (PRIMe)	Recruiting	No results available	PC	Dietary supplements (powdered formula containing whey protein and arginine, omega-3 fatty acids)	Canada	https://clinicaltrials.gov/ct2/show/NCT04549662
	Effects on Quality of Life with Zinc Supplementation in Patients with Gastrointestinal Cancer	Recruiting	No results available	PC, gastric, esophageal, liver and intrahepatic bile duct carcinoma	Dietary supplement (zinc)	U.S.	https://clinicaltrials.gov/ct2/show/NCT03819088
	Evaluation of Ocoxin-Viusid^®^ in Advanced Pancreatic Adenocarcinoma	Recruiting	No results available	PC, pancreatic diseases, digestive system neoplasms and diseases, endocrine system neoplasms and diseases	Dietary supplement (Ocoxin-Viusid^®^)	Cuba	https://clinicaltrials.gov/ct2/show/NCT03717298
	Survivorship Promotion in Reducing IGF-1 Trial (SPIRIT)	Completed	Has results	PC, breast, prostate, lung, colon, skin, endometrial, liver, rectal, kidney cancer and other solid malignant tumors	Changes in dietary intake	U.S.	https://clinicaltrials.gov/ct2/show/NCT02431676
	Prevention of Cancer-associated Malnutrition Through Oral Nutritional Supplements	Unknown	No results available	PC, hepatocellular carcinoma	Oral nutritional supplement (Fortimel Compact/Fortimel Compact Fiber, Nutricia)	Germany	https://clinicaltrials.gov/ct2/show/NCT02312674
**FMT**	Fecal Microbial Transplants for the Treatment of Pancreatic Cancer	Not yet recruiting	No results available	PDAC	FMT during colonoscopy and capsules	U.S.	https://clinicaltrials.gov/ct2/show/NCT04975217

IBD, inflammatory bowel disease; PC, pancreatic cancer; PDAC, pancreatic ductal adenocarcinoma; SOC, standard of care.

## Abbreviations

FMT	Fecal Microbial Transplantation
PanIN	Pancreatic Intraepithelial Neoplasia
PC	Pancreatic cancer
PDAC	Pancreatic ductal adenocarcinoma
SCFA	Short-chain fatty acids
